# Microbiota of the first‐pass meconium and subsequent atopic and allergic disorders in children

**DOI:** 10.1111/cea.14117

**Published:** 2022-03-03

**Authors:** Katja Kielenniva, Sofia Ainonen, Petri Vänni, Niko Paalanne, Marjo Renko, Jarmo Salo, Mysore V. Tejesvi, Tytti Pokka, Anna Maria Pirttilä, Terhi Tapiainen

**Affiliations:** ^1^ PEDEGO (Pediatrics, Dermatology, Gynecology, Obstetrics) Research Unit and Medical Research Center Oulu University of Oulu Oulu Finland; ^2^ Ecology and Genetics Faculty of Science University of Oulu Oulu Finland; ^3^ Genobiomics Ltd. Oulu Finland; ^4^ Department of Pediatrics and Adolescent Medicine Oulu University Hospital Oulu Finland; ^5^ Department of Pediatrics University of Eastern Finland Kuopio Finland; ^6^ Biocenter Oulu University of Oulu Oulu Finland

**Keywords:** 16S rRNA, allergic disorders, atopic eczema, cow's milk allergy

## Abstract

**Background:**

Some cohort studies have suggested that gut microbiota composition is associated with allergic diseases in children. The microbiota of the first‐pass meconium, which forms before birth, represents the first gut microbiota that is easily available for research and little is known about any relationship with allergic disease development.

**Objective:**

We investigated whether the bacterial composition of the first‐pass meconium is associated with the development of allergic diseases before 4 years of age.

**Methods:**

Prospective birth cohort study. Bacterial composition of first‐pass meconium was analysed using bacterial 16S rRNA gene amplicon sequencing. Atopic and allergic diseases were evaluated via online survey or telephone to age 4 years, based on the International Study of Asthma and Allergies in Childhood questionnaire.

**Results:**

During a 6‐week period in 2014, 312 children were born at the Central Finland Central Hospital. Meconium was collected from 212 at a mean of 8‐hour age. Outcome data at 4 years were available for 177 (83%) children, and 159 of these had sufficient amplification of bacterial DNA in meconium. Meconium microbiota composition, including diversity indices and relative abundances of the main phyla and genera, was not associated with subsequent atopic eczema, wheezing or cow's milk allergy. Principal components analysis did not identify any clustering of the meconium microbiomes of children with respect to wheezing or cow's milk allergy.

**Conclusions:**

We found no evidence that gut microbiota composition of first‐pass meconium is associated with atopic manifestations to age 4 years. However, larger studies are needed to fully exclude a relationship.

## Graphical abstract

Gut microbiota composition of the first stool after birth and the development of allergic diseases before 4 years of age were investigated in a population‐based cohort study of 212 infants. Atopic and allergic diseases were evaluated at 4 years of age and outcome data were available for 177 (83%) of the children. Meconium microbiota composition, including bacterial diversity indices and relative abundances of the main phyla and genera, was not associated with subsequent atopic eczema, wheezing or cow's milk allergy.

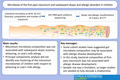

**Key messages**

- Some cohort studies have suggested that gut microbiota composition may be associated with allergic disease development.
- In this study, bacterial composition of first‐pass meconium was not associated with allergic disease development.
- Sample size was a limitation, so larger studies are needed to fully exclude a relationship.

## 1 INTRODUCTION

Early gut colonization is essential for the normal development and regulation of immune responses.^1^, ^2^ According to the environmental biodiversity hypothesis, a lack of biodiversity in the living environment leads to dysbiosis of the microbiota, impaired regulation of the development of immunological tolerance and inappropriate inflammatory responses.^3^, ^4^, ^5^, ^6^ Several cohort studies have reported an association between the human gut microbial composition in the 1st month of life and the subsequent risk of atopic disorders.^7^, ^8^, ^9^, ^10^, ^11^, ^12^

The microbiota of the first‐pass meconium, which forms before birth, represents the first gut microbiota that is easily available for research. Recent studies using the culture‐independent technique of next‐generation sequencing of the bacterial 16S ribosomal RNA (rRNA) gene have shown that the first‐pass meconium contains a diverse microbiota.^13^, ^14^, ^15^, ^16^ Maternal antibiotics and environmental biodiversity before birth have been associated with the microbial composition of the first‐pass meconium.^16^ Furthermore, maternal use of antibiotics during pregnancy has been linked with an increased risk of childhood asthma.^17^ Finally, the maternal microbiota during pregnancy has been reported to play a role in the development of a tolerogenic immune phenotype.^18^ A study with 20 newborn infants proposed an association between the meconium microbiota and subsequent respiratory problems.^14^ However, the clinical significance of the first‐pass meconium microbiota for the development of allergic diseases is largely unknown because the majority of large high‐quality cohort studies have not used the first‐pass meconium in their analyses.^10^, ^11^, ^12^

In this population‐based prospective cohort study of 212 consecutive newborn infants, we set out to investigate the gut microbial composition of the first stool after birth and the development of subsequent allergic diseases before 4 years of age.

## 2 METHODS

### 2.1 Study design and study population

Our study design was a prospective population‐based cohort study. We enrolled 212 consecutive near‐term and term infants born at the Central Finland Central Hospital, the only hospital that provided obstetric care in Central Finland between 3 February and 13 March 2014. All pregnant women during the study period were invited to participate in the study. Altogether, 312 children were born during the study period. The families of 218 newborn infants gave their informed consent, and the first stool sample was received from 212 infants. No other exclusion or inclusion criteria were used. The midwives collected the spontaneously evacuated, first‐pass meconium from the diaper of each newborn infant and placed the material into two sample tubes. Similar diapers and sample tubes were used throughout the study. All meconium samples were collected from the first stool after birth. The mean collection time was 8 h (range 0–32 h). The midwives recorded details about the pregnancies and deliveries. Throughout the study, routine antibiotic prophylaxis during caesarean section was not used. For this same cohort, we previously collected data on the maternal influence on the meconium microbiota^16^ and the association of meconium microbiota with infantile colic with a survey at 1 year^19^ and overweight at 3 years of age.^20^ Now, at 4 years of age, we conducted an active follow‐up of all participants regarding their atopic and allergic diseases. The study plan was reviewed and accepted by the Ethics Committee of the Central Finland Hospital District (1E/2014). All the families provided their written informed consent for the study.

### 2.2 Collection of clinical data

Detailed maternal and perinatal background characteristics were collected at the maternity ward at birth. The child's diet, including the duration of breastfeeding, was recorded in a survey at 1 year of age. When the children reached 4 years of age, all 212 families were first contacted via a letter containing a short survey about the children's allergies, asthma and other symptoms of atopic diseases. The families then received a link to an extended web‐based survey. Families who did not complete the web‐based survey were contacted via telephone. Our web‐based survey was based on the International Study of Asthma and Allergies in Childhood (ISAAC) questionnaire, with specific questions concerning allergies, asthma, other symptoms of atopic diseases and background factors (Table S1). The ISAAC questionnaire has previously been validated in the Finnish population.^21^, ^22^

### 2.3 Microbiota analyses

We previously reported the collection, storage and analysis of first‐pass meconium samples collected by midwives in the delivery room or perinatal wards.^16^ All the microbiota analyses were performed blinded to the infants’ clinical symptoms, and the parents were unaware of the microbiota results. We have previously reported fecal DNA extraction, amplification of the bacterial 16S rRNA genes and bioinformatics analyses.^16^, ^19^, ^20^ In brief, the Ion Torrent sequences were processed and analysed with QIIME 1.9.0 using state‐of‐the‐art procedures.^23^ The raw Ion Torrent data of the meconium and follow‐up fecal samples were deposited in the NCBI‐SRA with the accession number ‐SRP069890. We have previously reported the quality control analysis of the sample tubes, diapers, water and extraction kits.^16^, ^19^, ^20^ Before conducting the bioinformatics analyses and calculating the relative abundances, the known environmental contaminant *Rhodanobacter* was removed from the data set as in previous studies of the same cohort.^16^, ^19^, ^20^

### 2.4 Outcomes

The main outcome was the proportion of children with any atopic manifestation, i.e., atopic eczema, asthma, wheezing or cow's milk allergy. All outcomes were defined using the following questions that we retrieved from either the web‐based survey or telephone calls. The definition of atopic eczema diagnosed by a physician was based on responses to two questions: “Has the child ever had any itchy eczema?” and “Has a physician diagnosed the child with atopic eczema?” The definition of asthma was based on the response to the question, “Has your child's asthma been diagnosed by a physician?” The definition of wheezing was based on the parent‐reported response to the question, “Has your child ever had wheezing or whistling in the chest?” The definition of a cow's milk allergy was determined by the question, “Has your child ever had a cow's milk allergy diagnosed by a physician?” If this question was answered affirmatively, we asked whether the child had skin symptoms, gastrointestinal symptoms or both. These data were actively collected from families via telephone. Cow's milk allergy diagnoses were verified using the medical records of the patients treated in the hospital's outpatient clinic. Diagnoses were verified using the hospital's medical records of seven children. Cow's milk allergy diagnoses were made using the skin prick test (*n* = 1), the oral food challenge test (*n* = 3), both the skin prick test and oral food challenge test (*n* = 1) and gastrointestinal symptoms or skin symptoms together with specific immunoglobulin E (IgE) antibodies (*n* = 2). Seven other children were not diagnosed at the hospital's outpatient clinic; thus, their diagnoses were not confirmed. We report the answers to the web‐based survey (*n* = 134) that relate to the main outcome and gut microbiome results in supplementary file.

### 2.5 Statistical analyses

Before the study, we estimated the required sample size based on the first clinical microbiota studies, which showed altered gut microbiota composition in 12 infants with infantile colic compared to 12 controls.^24^ Thus, we estimated that the number of infants with subsequent atopic eczema, wheezing or cow's milk allergy would be sufficient for reasonable statistical power with a population‐based cohort of 150–200 children. We chose bacterial genera that have been associated with atopic eczema, allergies or wheezing for statistical comparisons to avoid coincidental findings.^8^, ^10^, ^11^, ^25^

We analysed the differences in bacterial diversity indices (Chao 1 and Shannon‐Weaver), the number of operational taxonomic units (OTUs) and the relative abundances of major bacterial phyla and genera with respect to later atopic eczema, wheezing and cow's milk allergy. In the analysis, samples with fewer than 1000 reads were considered to be samples that did not amplify sufficiently and were coded as zero for the relative abundance values and OTUs, i.e., the lowest possible value instead of a missing value. To compare the proportions of first‐pass meconium samples that did not amplify sufficiently, we used the standard normal deviation (SND) test on StatsDirect.

We used a t‐test to compare the mean number of sequence reads between children with and without later atopic eczema, wheezing, asthma and cow's milk allergy and furthermore, adjusted the analysis for the mode of delivery and sampling time (hours). We used the Mann–Whitney U test for all microbiota composition analyses with Bonferroni correction of the crude *p*‐values to compensate for multiple testing. The corrected *p*‐value was calculated by multiplying the crude *p*‐value by the number of comparisons if the crude *p*‐value was below .05. We performed a principal coordinate analysis (PCoA) to describe the intestinal microbiota at birth with respect to wheezing and cow's milk allergy. The principal coordinate analyses were performed using QIIME2 (2019.10) and the q2‐diversity plugin.^26^ The statistical analyses were performed with SPSS version 24 software (SPSS, Inc.).

## 3 RESULTS

### 3.1 Study population

**FIGURE 1:**
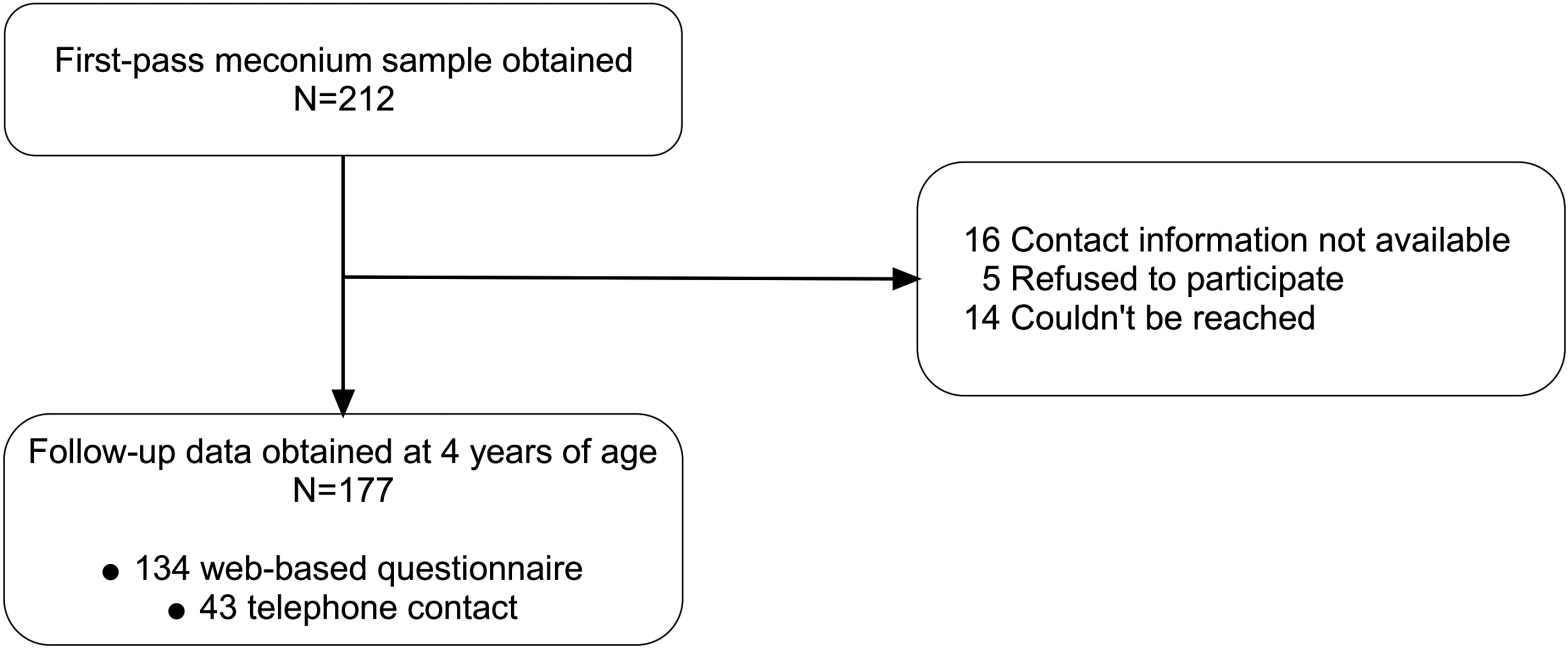
Flowchart of the study

Detailed maternal and perinatal background characteristics were collected from 212 children in the maternity ward at birth. At the age of 4 years, follow‐up data were available from 177 families (83%); 134 families completed a detailed web‐based survey about allergic and atopic diseases throughout life and 43 families responded to a letter survey or were interviewed via telephone (Table 1, Figure 1). Of the 177 families with follow‐up data available at 4 years of age, 140 completed the survey at 1 year of age, including information regarding the child's diet and duration of breastfeeding.

**TABLE 1:** Baseline characteristics of the whole original study population (*N* = 212) and those with follow‐up data (*n* = 177)

	Atopic eczema *n* = 50	Wheezing^a^ *n* = 25	Cow's milk allergy^b^ *n* = 14	All children with follow‐up data *n* = 177	Original study cohort *N* = 212
Gender					
Boy, *n* (%)	29 (58)	14 (54)	10 (71)	96 (54)	114 (54)
Girl, *n* (%)	21 (42)	12 (46)	4 (29)	81 (46)	98 (46)
Mother's education level					
Comprehensive school, *n* (%)	2 (4.0)	0 (0)	0 (0)	7 (4)	14 (7)
High school, *n* (%)	1 (2.0)	0 (0)	0 (0)	14 (8)	17 (8)
Vocational school, *n* (%)	15 (30)	12 (46)	3 (21)	57 (32)	72 (34)
University of applied sciences, *n* (%)	19 (38)	8 (31)	7 (50)	53 (30)	60 (28)
University, *n* (%)	13 (26)	6 (23)	4 (29)	45 (26)	48 (23)
Mode of delivery					
Vaginal, *n* (%)	37 (74)	17 (65)	11 (79)	140 (79)	172 (81)
Caesarean section, *n* (%)	13 (26)	9 (35)	3 (21)	37 (21)	40 (19)
Gestational age in weeks (range)	39.4 (36.6–42.3)	39.2 (37.1–42)	39.7 (36.6–42.3)	39.7 (36.6–42.4)	39.6 (35.4–42.4)
Number of siblings at birth					
None, *n* (%)	19 (39)	9 (36)	8 (57)	64 (37)	72 (34)
One, *n* (%)	13 (26)	6 (24)	3 (21.5)	52 (30)	67 (32)
Two or more, *n* (%)	17 (34)	10 (40)	3 (21.5)	59 (34)	70 (33)
Mean birth weight in grams (SD)	3525 (538)	3369 (596)	3443 (597)	3561 (517)	3553 (494)
Maternal antibiotics during pregnancy, *n* (%)	11 (22)	7 (27)	3 (21)	36 (20)	37 (18)
Antibiotics during delivery, *n* (%)^c^	17 (34)	8 (31)	5 (36)	51 (29)	61 (29)
Child's antimicrobials in neonatal ward, *n* (%)^d^	0 (0)	1 (3.8)	0 (0)	4 (2.3)	4d (1.9)
Mother's atopic eczema^e^, *n* (%)	16 (37)	6 (23)	4 (29)	26 (20)	NA^g^
Father's atopic eczema^e^, *n* (%)	9 (21)	7 (27)	1 (7.1)	25 (19)	NA
Mother's asthma^e^, *n* (%)	4 (9.3)	3 (12)	3 (21)	10 (7.5)	NA
Father's asthma^e^, *n* (%)	5 (12)	2 (7.7)	3 (21)	10 (7.5)	NA
Maternal smoking during pregnancy^e^, *n* (%)	3 (7.0)	1 (3.8)	0 (0)	5 (3.7)	NA
Parental current smoking^e^, *n* (%)	7 (17)	6 (23)	2 (15)	26 (20)	NA
Mean duration of breastfeeding^f^ in months (SD)	10.5 (3.3)	10.6 (2.9)	11.2 (1.2)	9.2 (3.7)	NA
Mean duration of exclusive breastfeeding^f^ in months (SD)	4.6 (2.4)	4.8 (1.9)	5.3 (0.87)	3.8 (2.2)	NA

^a^ Information about wheezing was obtained for only 134 children, whose families completed the online survey. The information about atopic eczema and cow's milk allergy was obtained from all of the 177 children.

^b^ Of the 14 children with cow's milk allergy, four had gastrointestinal symptoms, two had dermatological symptoms and eight had both gastrointestinal and dermatological symptoms. Cow's milk allergy was diagnosed by a physician.

^c^ For 61 neonates exposed to antibiotics during delivery: Cefuroxime (*n* = 31), Benzylpenicillin (*n* = 28), Piperacillin/Tazobactam (*n* = 2).

^d^ Benzylpenicillin and tobramycin.

^e^ Information about a parent's atopic eczema, asthma and smoking was obtained for only 134 children, whose families completed the online survey.

^f^ Information about breastfeeding was collected via clinical survey when the children were 1 year of age and was available for 140 of the families who had also completed the survey at 4 years of age. Exclusive breastfeeding was defined as the situation in which the child was fed neither solid foods nor formula.

^g^ NA, Not available.

### 3.2 Meconium samples and clinical outcomes

#### 3.2.1 Number of sequences

In total, 72 children had at least one atopic manifestation. In total, 50 children (8%) had atopic eczema, 4 (2%) had asthma, 26 (19%) had later wheezing and 14 (8%) had cow's milk allergy. In total, 18 of the first‐pass meconium samples (10%), with follow‐up data available (*n* = 177), exhibited insufficient amplification in PCR, suggesting a very low amount of bacterial DNA was present in the samples.

In the analysis adjusted for the mode of delivery and sampling time after birth, children with any atopic manifestation (*n* = 69)—i.e., atopic eczema, asthma, wheezing or cow's milk allergy,—had fewer sequence reads in the meconium samples compared to those without any atopic manifestations (*n* = 67) (11,133 [SD 14,834] vs. 17,546 [SD 15,228], *p* = .02, 95% CI [−11,122 to −918], respectively) (Table 2). Sampling time was not recorded for six children; thus, those samples were removed from the adjusted analysis. There were no differences in the proportions of samples that exhibited insufficient amplification in PCR in children with any atopic disease or wheezing and in those who remained healthy in this respect (Table 3).

**TABLE 2:** Association of sequence reads in the meconium samples with any later atopic manifestation, atopic eczema, wheezing and cow's milk allergy – analysis adjusted for mode of delivery and sampling time

	Mean number of raw bacterial sequences (SD)	*p*‐value^a^	95% CI of the difference
Any atopic manifestation^b^, *n* = 69	11,133 (14,834)	.021	−11,122 to −918
No atopic manifestation, *n* = 67	17,546 (15,228)		
Atopic eczema, *n* = 47	13,311 (15,916)	.67	−6278 to 4028
No atopic eczema, *n* = 124	14,706 (14,896)		
Wheezing^c^, *n* = 26	9075 (12,642)	.051	−13,149 to 19,976
No wheezing, *n* = 103	15,886 (15,763)		
Cow's milk allergy, *n* = 12	5048 (10,182)	.03	−19,031 to −1242
No cow's milk allergy, *n* = 158	15,022 (15,302)		

^a^ The analysis was adjusted for mode of delivery and sampling time of the meconium samples after birth. The sampling time was recorded for only 171 of the children.

^b^ Any manifestation of asthma, wheezing, atopic eczema or cow's milk allergy.

^c^ Information about wheezing was obtained for only 134 children, whose families completed the online survey.

**TABLE 3:** Association of amplification products in the meconium samples any later atopic manifestation, atopic eczema, wheezing and cow's milk allergy

	Samples without product^a^ *n* = 18	Samples with product *n* = 159	*p*‐value^b^	95% CI of difference
Any atopic manifestation^c^, *n* = 72	8 (11%)	64 (89%)	.59	−8.2 to 13
No atopic manifestation, *n* = 69	6 (8.7%)	63 (91.3%)		
Atopic eczema, *n* = 50	7 (14%)	43 (86%)	.20	−4.1 to 18
No atopic eczema, *n* = 127	11 (8.7%)	116 (91.3%)		
Wheezing^d^, *n* = 26	3 (12%)	23 (88%)	.52	−9.3 to 19
No wheezing, *n* = 108	11 (10%)	97 (90%)		
Cow's milk allergy, *n* = 14	4 (29%)	10 (71%)	.02	2.2 to 46
No cow's milk allergy, *n* = 162	14 (8.6%)	148 (91.4%)		

^a^ This suggests low bacterial DNA concentration in the sample.

^b^ A standard normal deviation test for comparing proportions was used for comparisons.

^c^ Any manifestation of asthma, wheezing, atopic eczema or cow's milk allergy.

^d^ Information about wheezing was obtained for only 134 children, whose families completed the online survey.

#### 3.2.2 Atopic eczema

In the analysis adjusted for delivery mode and sampling time after birth, the mean number of sequences in the meconium microbiota was lower in children who were diagnosed with atopic eczema (*n* = 47) compared to those who were not (*n* = 124), but the difference was not statistically significant (13,311 [SD 15,916] vs. 14,706 (14,896), *p* = .67, 95% CI [−6278 to 4028]) (Table 2).

There were no statistical differences between the gut microbiota composition, including bacterial diversity indices and relative abundances of the main phyla and genera, of the first stool after the birth of children with physician‐diagnosed atopic eczema and those without it in the analysis corrected for multiple testing (Table 4).

**TABLE 4:** Composition of gut microbiome in the first stool after birth in children with atopic manifestations and those who remained healthy

	Atopic eczema *n* = 50 (28%)	No atopic eczema *n* = 127 (72%)	Corrected *p*‐value^a^	Wheezing *n* = 26	No wheezing *n* = 108	Corrected *p*‐value^a^	Cow's milk allergy, *n* = 14	No cow's milk allergy, *n* = 162	Corrected *p*‐value^a^
Bacterial diversity indices									
Shannon diversity index (SD)	5.7 (1.5)	5.5 (1.69)	.49	5.8 (1.3)	5.6 (1.6)	.75	5.9 (1.4)	5.5 (1.6)	.42
Chao1 (SD)	330 (221)	324 (221)	.73	322 (211)	320 (221)	.70	329 (206)	325 (222)	.74
OTUs^b^ (SD)	178 (129)	181 (126)	.93	176 (123)	203 (117)	.95	153 (137)	182 (126)	.45
Phyla									
Firmicutes, mean %^c^ (SD)	35 (30)	44 (33)	.14	34 (30)	43 (33)	.27	23 (28)	43 (32)	.24^a^
Proteobacteria, mean % (SD)	31 (34)	29 (34)	.70	36 (35)	27 (34)	.35	38 (42)	29 (33)	.77
Bacteroidetes, mean % (SD)	15 (20)	15 (22)	.81	13 (20)	16 (22)	.90	9.9 (20)	15 (22)	.25
Actinobacteria, mean % (SD)	1.0 (3.7)	0.71 (1.8)	.57	1.1 (2.2)	0.6 (1.5)	.12	0.63 (1.8)	0.80 (2.5)	.52
Genera and species									
*Bacteroides* spp., mean % (SD)	12 (18)	13 (20)	.83	11 (19)	13 (20)	.90	8.6 (17)	13 (20)	.29
*Staphylococcus* spp., mean % (SD)	12 (25)	13 (25)	.63	16 (27)	13 (24)	.59	2.4 (5.4)	14 (25)	.70^a^
*Streptococcus* spp., mean % (SD)	4.0 (9.3)	5.1 (9.4)	.45	6.2 (8.4)	4.6 (8.7)	.58	3.3 (4.8)	5.0 (9.7)	.60
*Enterococcus* spp., mean % (SD)	2.3 (14)	5.6 (21)	.11	0.38 (0.85)	5.3 (21)	.56	0.08 (0.20)	5.1 (20)	.28
*Lactobacillus* spp., mean % (SD)	2.9 (13)	4.1 (13)	.51	1.2 (2.9)	4.3 (14)	.27	7.4 (24)	3.5 (11)	.46
*Faecalibacterium* spp., mean % (SD)	1.2 (2.3)	1.3 (2.3)	.72	0.89 (2.0)	1.3 (2.3)	.59	0.90 (2.2)	1.3 (2.3)	.68
*Clostridium* spp., mean % (SD)	0.13 (0.86)	1.4 (10)	.52	0.003 (0.02)	1.6 (11.3)	.59	0% (0)	1.1 (9.3)	.39
*Prevotella* spp., mean % (SD)	0.28 (1.2)	0.09 (0.28)	.32	0.02 (0.05)	0.15 (0.82)	.26	0.03 (0.08)	0.15 (0.72)	.56
*Veillonella* spp., mean % (SD)	0.09 (0.25)	0.10 (0.22)	.84	0.06 (0.21)	0.10 (0.25)	.24	0.06 (0.16)	0.10 (0.23)	.45
*Bifidobacterium* spp., mean % (SD)	0.003 (0.02)	0.002 (0.02)	.85	0.01 (0.03)	0.002 (0.02)	.35	N/A	0.003 (0.02)	1.0
*Bacteroides fragilis*, mean % (SD)	2.2 (3.5)	3.3 (9.5)	.78	1.9 (3.6)	3.6 (10)	.67	1.6 (3.2)	3.1 (8.6)	.41
*Faecalibacterium prausnitzii*, mean % (SD)	0.87 (1.7)	0.84 (1.5)	.80	0.62% (1.4)	0.87% (1.5)	1.0	0.67% (1.6)	0.86% (1.6)	.73
*Escherichia coli*, mean % (SD)	0% (0)	0.40 (4.4)	.56	0% (0)	0.01% (0.08)	.64	0% (0)	0.31 (3.9)	1.0

^a^ Bonferroni correction was used if crude *p*‐value was <.05.

^b^ OTU: operational taxonomic unit.

^c^ Mean relative abundance.

#### 3.2.3 Asthma and wheezing

We used wheezing in the main analysis because the number of children with physician‐diagnosed asthma was low (*n* = 4). In total, 26 children (19%) had parent‐reported wheezing before 4 years of age.

In the analysis adjusted for delivery mode and sampling time after birth, the mean number of sequences in the first stool after birth was lower in children with later wheezing (*n* = 26) compared to those without (*n* = 103) (9075 [SD 12,642] vs. 15,886 [SD 15,763], *p* = .05, 95% CI [−13,149 to 19,976], respectively) (Table 2). There were no differences between the proportions of samples that exhibited insufficient amplification in PCR in children with wheezing and those who remained healthy in this respect (Table 3).

There were no differences in gut microbiota composition between children with parent‐reported wheezing and those who remained healthy in the analysis corrected for multiple testing (Table 4). There was no clustering in the PCoA of the meconium microbiomes of children with later wheezing (*n* = 26) (Figure 3A).

#### 3.2.4 Cow's milk allergy

**FIGURE 2:**
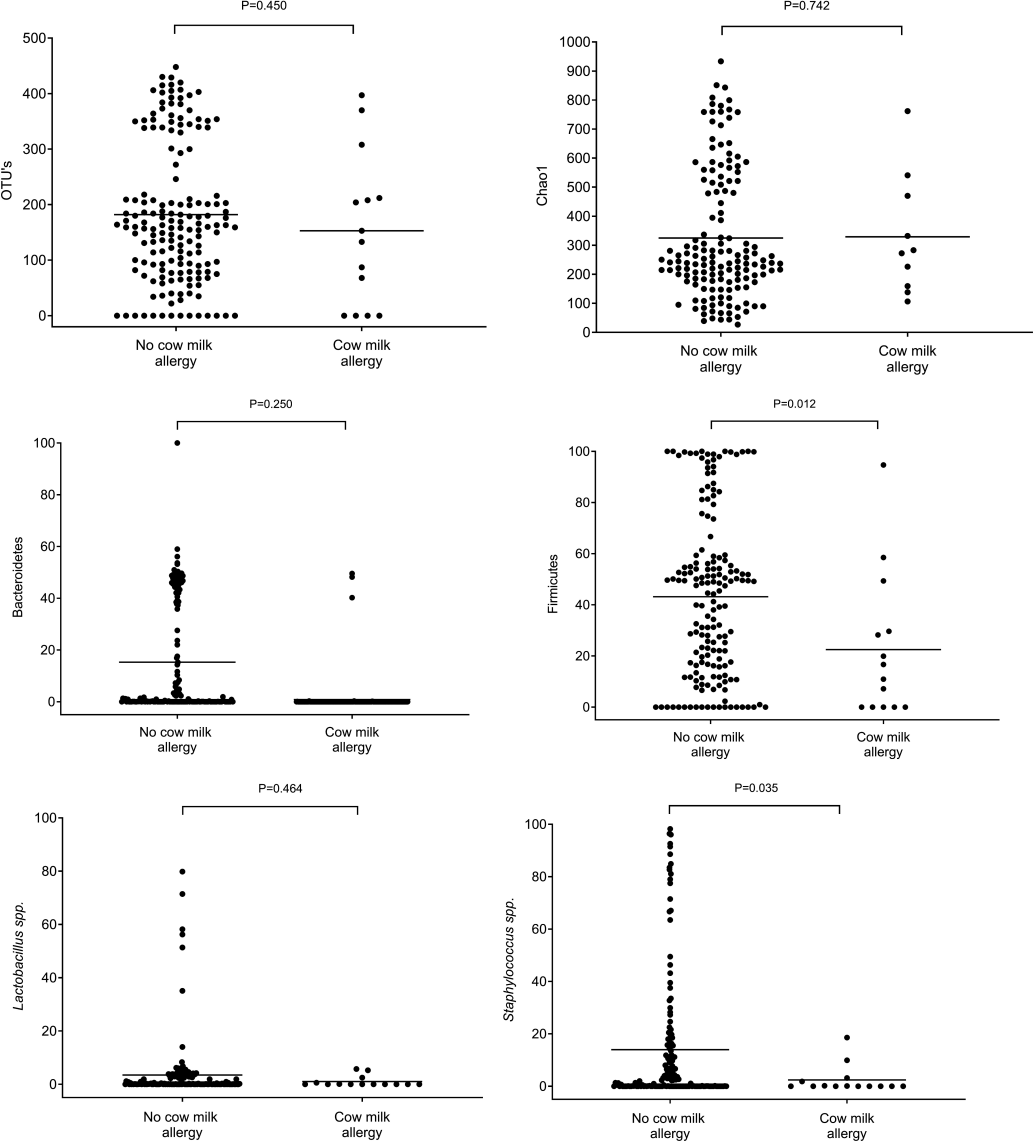
Number of OTUs, Chao1 diversity index, and relative abundances of the phyla Bacteroidetes and Firmicutes and the genera Lactobacillus and Staphylococcus in children with and without cow’s milk allergies

**FIGURE 3:**
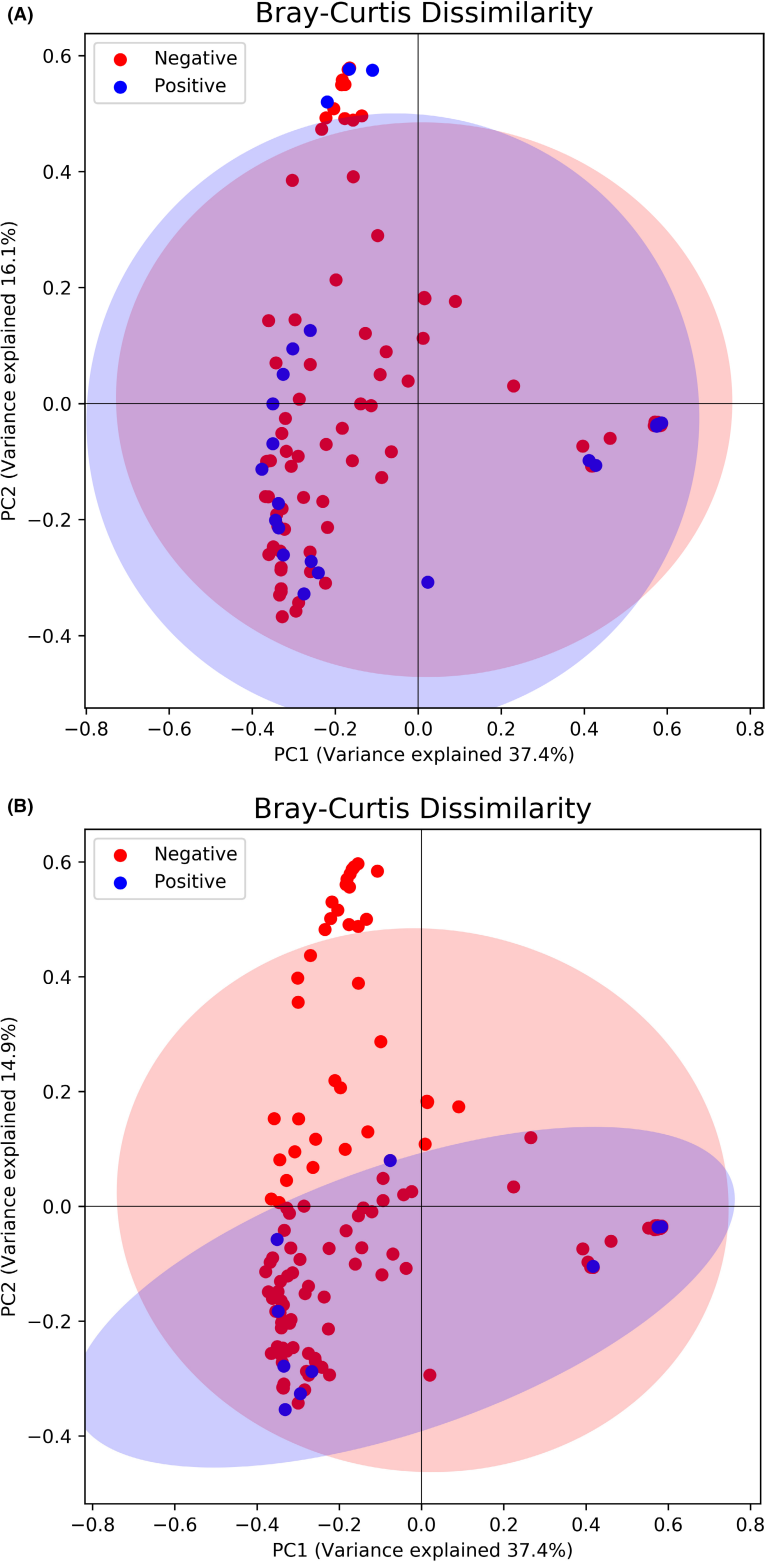
Principal co‐ordinate analysis (PCoA) of meconium microbiota and A) wheezing, *p* = .055 and B) cow’s milk allergy, *p* = .31

In the analysis adjusted for multiple testing, there was no difference in the gut microbiota composition between children with later cow's milk allergy and those who remained healthy (Table 4, Figure 2). The PCoA showed no differences between the meconium microbiota of children with later cow's milk allergy and those who remained healthy (Figure 3B)

#### 3.2.5 Other questions in the survey

Data obtained from the full survey are reported in Tables S1–S5. As the main outcomes were not statistically significant in the analyses, further statistical analyses were not performed for other outcomes received from the full web‐based survey (Tables S2–S5).

## 4 DISCUSSION

In this prospective cohort study of 212 consecutive newborn infants, the gut microbiota composition of the first‐pass meconium was not associated with later atopic manifestations in children. The exact timing of the first bacterial contact may require further investigation, since infants with later atopic manifestation appeared to have fewer raw bacterial sequences in the first stool.

Earlier paediatric cohorts have evaluated the development of allergic diseases and gut microbiota. However, unlike our study, they did not use the first‐pass meconium for their analyses, and they started follow‐ups at the age of 1 week or later (Table 5).^7^, ^8^, ^9^, ^11^, ^12^, ^25^, ^27^, ^28^, ^29^ These studies reported differences in the colonization process of the gut by staphylococci, enterococci, bifidobacteria and *Clostridium difficile*, as well as reduced bacterial diversity in children with allergic sensitization and eczema.^7^, ^8^, ^12^, ^27^, ^28^, ^29^ In the COPSAC cohort study of 648 children, a higher relative abundance of *Veillonella* and a lower relative abundance of *Roseburia*, *Alistipes* and *Flavonifractor* in the intestinal microbiota at 1 year of age were associated with an increased risk of asthma at 5 years of age.^10^ Another study investigating the role of early bacterial flora and later allergic sensitization showed that lower bacterial diversity in the gut microbiota at the ages of 1 month and 1 year was associated with allergic sensitization assessed via serum IgE levels, skin prick tests and allergic rhinitis.^11^ In a Consortium of Food Allergy (CoFAR) observational study of food allergies, of 226 children with a cow's milk protein allergy, the children whose milk allergy was resolved by 8 years of age had Clostridia and Firmicutes enriched in their intestinal microbiota at 3–16 months of age.^30^ In animal models, perinatal antibiotic exposure has been associated with altered microbiota and later responses to allergens.^31^ In this study of first‐pass meconium microbiota, gut microbiota composition was not associated with later atopic eczema, wheezing or cow's milk allergy in children. The lower number of reads in the first stool samples appeared to be associated with later allergic diseases in the present study, which may reflect slower bacterial colonization of the infant gut. This finding requires further investigation.

**TABLE 5:** Findings from some previous prospective studies about the association of early intestinal microbiota with later allergies, asthma or atopic eczema

Author	Study design	Study population	Method used	Fecal sample collection	Significant results
Abrahamsson et al. 2012^8^	Prospective cohort study	Infants with IgE‐associated eczema (*n* = 20) and controls (*n* = 20)	16S rRNA gene sequencing	1 week, 1 month and 12 months of age	Lower bacterial diversity, lower Bacteroidetes and *Bacteroides* at 1 month and lower Proteobacteria at 12 months in infants with eczema
Abrahamsson at al. 2014^7^	Prospective cohort study	Infants (*N* = 47) followed until 7 years of age	16S rRNA gene sequencing	1 week, 1 month and 12 months of age	Children with asthma had lower bacterial diversity at 1 week and 1 month
Adlerberth et al. 2007^9^	3 birth cohorts in Göteborg, London and Rome	Infants (*N* = 324) followed until 18 months of age	Culture	3, 7, 14 and 28 days and 2, 6 and 12 months of age	Atopic eczema was not associated with any particular bacterial group
Arrieta et al. 2015^25^	prospective cohort study	Infants (*N* = 319) in the Canadian Healthy Infant Longitudinal Development (CHILD) study followed until 5 years of age	16s rRNA gene sequencing	3 months and 1 year of age	Decreased *Lachnospira*, *Veillonella*, *Faecalibacterium* and *Rothia* at 3 months in children with later asthma
Bisgaard et al. 2011^11^	Prospective cohort study	Children (*N* = 411) with high risk of allergic diseases	16S rRNA gene sequencing and culture	1 month and 12 months of age	Bacterial diversity at 1 and 12 months was inversely associated with the risk of allergic sensitization and allergic rhinitis but not with asthma or atopic eczema
Björkstén et al. 2001^27^	Prospective cohort study	Infants in Estonia (*n* = 24) and Sweden (*n* = 20) followed until 2 years of age	Culture	5 to 6 days and 1, 3, 6 and 12 months of age	Children with allergies were less often colonized with enterococci at 1 month and bifidobacteria at 1 year, had higher counts of clostridia at 3 months and staphylococcus at 6 months and lower *Bacteroides* at 1 year
Ismail et al. 2012^28^	Prospective cohort study	Infants (*N* = 98) with high risk of allergic diseases	16S rRNA gene sequencing	1 week of age	Infants with eczema at 1 year of age had lower microbial diversity at 1 week of age
Penders et al. 2007^12^	Prospective cohort study	Infants (*N* = 957) participating the KOALA Birth Cohort Study	Real‐time PCR	1 month of age	Colonization with *E*.*coli* and *C*. *difficile* was associated with higher risk of later atopic eczema
Sjögren et al. 2009^29^	Prospective cohort study	Infants (*N* = 47) followed until 5 years of age	Real‐time PCR	1 week, 1 month and 2 months of age	Infants with allergy were less often colonized with certain lactobacilli species, *Bifidobacterium adolescentis* and *C*. *difficile* during first 2 months after birth
Stokholm et al. 2018^10^	Prospective cohort study	Infants (*N* = 690) participating the COPSAC birth cohort study	16s rRNA gene sequencing	1 week, 1 month and 1 year of age	In children born to asthmatic mothers, asthma at 5 years was positively associated with *Veillonella* and negatively with *Faecalibacterium*, *Bifidobacterium*, *Roseburia*, *Alistipes*, *Ruminococcus* and *Dialister* at 1 year

The first stool after birth, i.e., the first‐pass meconium, is the first easily available sample for gut microbiota research in infants. Several recent studies have reported diverse microbiota in the first‐pass meconium, both in vaginally delivered children and in those born via caesarean section, suggesting that the presence of bacterial DNA in the gut may start in utero or very early in the perinatal period.^13^, ^14^, ^16^ In our study, newborn infants with later allergic diseases often had less bacterial DNA in the first stool after birth than those who remained healthy. This finding suggests that the dynamics of very early gut colonization may play a role in the subsequent risk of allergic diseases. In animal models, the maternal microbiota may drive the postnatal innate immune development of offspring.^32^, ^33^ Interestingly, the administration of the probiotic *Lactobacillus rhamnosus* has been shown to successfully reduce atopic eczema in children when mothers receive probiotic products during pregnancy.^34^ Thus, maternal diet, living environment and gut microbiota may be important factors in the development of the infant's immune system.^35^ Accordingly, our study emphasizes the need for future studies on the role of microbial contacts during the fetal and perinatal periods in the pathogenesis of allergic diseases.

The strength of our study was its excellent design. In this prospective population‐based cohort study, the response rate was high at 4 years of age. To our knowledge, this is the first study to investigate the associations between allergic diseases and the gut microbiota composition of the first stool. The earlier reported associations observed between gut colonization and allergic diseases have been reported in cohorts with first samples collected between the ages of 1 week and 1 month.

The present study has some limitations. We were unable to verify cow's milk allergy diagnoses using medical records for all patients, and there could be significant variations in how the diagnoses were made. Furthermore, the prevalence of cow's milk allergy was relatively high (8%) in this study compared to earlier studies that reported that the incidence of challenge‐proven cow's milk allergy is 0.54%.^36^ This may reflect the variation in the diagnostic criteria, even though all diagnoses were made by physicians in the present study. In an unselected study population, the number of subjects with a specific disease is low. Due to the limited sample size, we did not analyse the impact of maternal microbiota, the living environment during pregnancy or other perinatal factors on the outcomes in this study. Finally, the limited amount of DNA in the first stool after birth makes microbiota analysis challenging. The genus *Rhodanobacter* was recognized as a contaminant and removed from the data set before the bioinformatics analyses.

In this population‐based cohort study, the gut microbiota composition of the first‐pass meconium was not associated with later atopic manifestations in children. Infants with later atopic manifestations appeared to have a limited amount of bacterial DNA in the first stool after birth, based on the low number of raw bacterial sequences in the samples compared to those who remained healthy. The exact timing of the first bacterial contact may require further investigation in the pathogenesis of allergic diseases.

## CONFLICT OF INTEREST

Katja Kielenniva, MD, has nothing to disclose. Sofia Ainonen, BM, has nothing to disclose. Petri Vänni, MSc, has nothing to disclose. Niko Paalanne, MD, PhD, has nothing to disclose. Marjo Renko, MD, PhD, has nothing to disclose. Jarmo Salo, MD, PhD, has nothing to disclose. Mysore V. Tejesvi, PhD, has nothing to disclose. Tytti Pokka, MSc, has nothing to disclose. Anna Maria Pirttilä, PhD, has nothing to disclose. Terhi Tapiainen, MD, PhD, has nothing to disclose.

## AUTHOR CONTRIBUTIONS

All the authors revised the manuscript for intellectual content, approved the final manuscript as submitted and agreed to be accountable for all aspects of the work. Katja Kielenniva, MD, drafted the data collection survey and spreadsheet, collected the clinical data, performed and interpreted the data analyses and wrote the first draft of the manuscript. Sofia Ainonen, BM, collected the clinical data. Petri Vänni, MSc, performed the bioinformatics analyses. Niko Paalanne, MD, PhD, wrote the research plan, organized the collection of stool samples and interpreted both the microbiota data and clinical data. Marjo Renko, MD, PhD, created the overall study design and planned the data analyses. Jarmo Salo, MD, PhD, assisted in developing and writing the research plan and interpreted the data. Mysore V. Tejesvi, PhD, performed 16S rRNA analyses and bioinformatics analyses and was responsible for the quality of the work in the research laboratory. Tytti Pokka, MSc, planned the statistical analyses and interpreted the data. Anna Maria Pirttilä, PhD, contributed to designing the study and analysing and interpreting the microbiota data. Terhi Tapiainen, MD, PhD, was the principal investigator and was involved in designing the study and analysing and interpreting the microbiota data and statistical data.

## ETHICAL STATEMENT

The research plan was accepted by the Ethics Committee of the Central Finland Hospital District in Jyväskylä, Finland.

## Supporting information

Table S1‐S5

## Data Availability

The raw Ion Torrent data of meconium and follow‐up fecal samples were deposited in the NCBI‐SRA with the accession number ‐SRP069890.
