# Supplementary material for: Microbiota of the first‐pass meconium and subsequent atopic and allergic disorders in children
Source: Clin Exp Allergy. 2022 Mar 3;52(5):684–96. doi: 10.1111/cea.14117 (PMC9314137; doi:10.1111/cea.14117)
Supplement: Supplementary file 1 — Table S1‐S5 [file CEA-52-684-s001.docx]

**Table S1.** Web-based survey was based on The International Study of Asthma and Allergies in Childhood (ISAAC) questionnaire with specific questions concerning allergy, asthma and other atopic symptoms, and background factors.

**Additional Questions not included to the ISAAC questionnaire**

1. Does your family have pets at home? Yes__ No__
2. Did mother of the child smoke during pregnancy? Yes__ No__
3. At the moment, does either of the parents smoke? Yes__ No__
4. At any time of life, does mother of the child have or have previously had any of the following diagnoses made by physician?
   1. Asthma Yes__ No__
   2. Allergic Rhinitis Yes__ No__
   3. Food allergy of any kind Yes__ No__
   4. Atopic eczema Yes__ No__
5. At any time of life, does father of the child have or have previously had any of the following diagnoses made by physician?
   1. Asthma Yes__ No__
   2. Allergic Rhinitis Yes__ No__
   3. Food allergy of any kind Yes__ No__
   4. Atopic eczema Yes__ No__
6. At any time of life, does any of the siblings of the child have or have previously had any of the following diagnoses made by physician?
   1. Asthma Yes__ No__
   2. Allergic Rhinitis Yes__ No__
   3. Food allergy of any kind Yes__ No__
   4. Atopic eczema Yes__ No__
7. Did you travel abroad during pregnancy? Yes__ No__
   1. If you stated yes, did you travel to Europe Yes__ No__
   2. If you stated yes, did you travel outside Europe? Yes__ No__
   3. Please, state the countries you visited during pregnancy
8. Did your child travel abroad during the first year of life? Yes__ No__
   1. If you stated yes, did you travel to Europe Yes__ No__
   2. If you stated yes, did you travel outside Europe? Yes__ No__
   3. Please, state the countries you visited during pregnancy
9. How many siblings does your child have?
10. How many children do live in your family in addition to the siblings of the child?
11. At any time of life, does your child have or has previously had asthma diagnosis made by physician?
    1. If you stated yes, in which age the diagnosis was made?
    2. Which asthma medication your child has used?
    3. Does your child currently use medication for asthma?
    4. In the last 12 months, has your child used regularly inhaled cortisone due to asthma?
12. At any time of life, has your child been diagnosed with allergic rhinitis by physician?
    1. If you stated yes, in which age the diagnosis was made?
13. During the two first years of life, did your child have wheezing associated to respiratory infection? Yes__ No__
14. In last 24 months, has your child had wheezing associated to respiratory infection?

Yes__ No__

1. At any time of life, has your child visited a physician due to respiratory infection associated wheezing? Yes__ No__
   1. If you stated yes, has your child been medicated due to wheezing at appointment?

Yes__ No__

- 1. During lifetime, how many infection-associated wheezing episodes has your child had?

1. At any time of life, has your child been diagnosed with atopic eczema by physician?

Yes__ No__

1. Does your child have eczema at the moment? Yes__ No__

1. If you stated yes, how do you treat the eczema? No treatment [ ], Moisturizing lotion [ ], Lotion containing cortisone, less than one week every month [ ], Lotion containing cortisone, more than one week every month [ ], Other lotions (such as tacrolimus) [ ]
2. At any time of life, has your child been diagnosed with cow’s milk allergy by physician?
3. If you stated yes, does the allergy lead to: skin symptoms [ ], intestinal symptoms [ ], both []
4. At any time of life, has your child been diagnosed with any other food allergy by physician?
   1. If you stated yes, for which food?
   2. How was the diagnosis made?

**Questions adapted from the ISAAC questionnaire**

1. Has your child ever had wheezing or whistling in the chest at any time in the past? Yes__ No__
2. If you stated yes, has your child had wheezing or whistling in the chest in the last 12 months? Yes__ No__
3. How many attacks of wheezing has your child had in the last 12 months? None [ ] 1 to 3 [ ] 4 to 12 [ ] More than 12 [ ]
4. In the last 12 months, how often, on average, has your child's sleep been disturbed due to wheezing? Never woken with wheezing [ ] Less than one night per week [ ] One or more nights per week [ ]
5. In the last 12 months, has your child's chest sounded wheezy during or after exercise?

Yes__ No__

1. In the last 12 months, has your child had a dry cough at night, apart from a cough associated with a cold or a chest infection? Yes__ No__
2. Has your child ever had a problem with sneezing, or a runny, or a blocked nose when he/she DID NOT have a cold or the flu? Yes__ No__
3. In the past 12 months, has your child had a problem with sneezing, or a runny, or a blocked nose when he/she DID NOT have a cold or the flu? Yes__ No__
4. In the past 12 months, has this nose problem been accompanied by itchy-watery eyes?

Yes__ No__

1. In which of the past 12 months did this nose problem occur? (please tick any which apply) January [ ] February [ ] March [ ] April [ ] May [ ] June [ ] July [ ] August [ ] September [ ] October [ ] November [ ] December [ ]
2. Has your child ever had an itchy rash which was coming and going for at least 6 months?

Yes__ No__

1. Has this itchy rash at any time affected any of the following places: the folds of the elbows, behind the knees, in front of the ankles, under the buttocks, or around the neck, ears or eyes?

Yes__ No__

1. At what age did this itchy rash first occur? Under 2 years [ ] Age 2–4 [ ] Age 5 or more [ ]
2. Has this rash cleared completely at any time during the last 12 months? Yes__ No__
3. Has your child ever had eczema? Yes__ No__

**Table S2.** Data regarding other variables in the questionnaire that are related to the diagnosis of atopic eczema made by physicians used in the main primary analysis.

|  | **OTU’s (SD)** | **Number of reads (SD)** | **Bacteroidetes,**  **mean % (SD)** | **Firmicutes, mean % (SD)** | **Proteobacteria, mean % (SD)** | **Bacteroides spp., mean % (SD)** | **Lactobacillus spp., mean % (SD)** | **Staphylococcus spp., mean % (SD)** |
| --- | --- | --- | --- | --- | --- | --- | --- | --- |
| Atopic eczema at any time (self reported) |  |  |  |  |  |  |  |  |
| Yes, N = 53 | 181 (128) | 12,887 (15,891) | 14% (20) | 38% (31) | 32% (35) | 12% (18) | 3.0% (12) | 13% (24) |
| No, N = 68 | 172 (126) | 14,211 (14,361) | 15% (21) | 44% (34) | 27% (34) | 12% (19) | 3.8% (13) | 15% (26) |
| Itchy rash for at least 6 months |  |  |  |  |  |  |  |  |
| Yes, during the last year,  N = 31 | 188 (125) | 14,760 (16,315) | 14% (20) | 45% (32) | 30% (35) | 11% (18) | 4.4% (16) | 17% (30) |
| Yes, but not during the last year, N = 15 | 151 (142) | 9,630 (14,292) | 13% (21) | 26% (28) | 28% (37) | 11% (19) | 1.0% (1.7) | 4.9% (9.4) |
| No, N = 88 | 179 (125) | 15,367 (15,187) | 16% (23) | 42% (33) | 29% (34) | 13% (21) | 3.9% (13) | 14% (23) |
| Itchy rash in the folds of the elbows, behind the knees, in front of the ankles, under the buttocks, or around the neck, ears or eyes |  |  |  |  |  |  |  |  |
| Yes, N =35 | 188 (129) | 12,502 (15,165) | 16% (21) | 37% (31) | 32% (36) | 13% (19) | 4.1% (15) | 10% (22) |
| No, N= 79 | 173 (123) | 14,834 (15,079) | 15% (22) | 43% (34) | 29% (34) | 11% (20) | 3.3% (12) | 17% (26) |

Number of participants with full data (*N* = 134) is lower than that of those variables used in the primary analysis (*N* = 177), because the outcomes used in the primary analysis were actively collected with telephone contacts if families did not respond to the full survey. Number of subjects with specific answers may differ due to lacking answers.

**Table S3.** Data regarding other variables in the questionnaire that are related to the diagnosis of asthma and parent-reported wheezing used in the main primary analysis.

|  | **OTU’s (SD)** | **Number of reads (SD)** | **Bacteroidetes,**  **mean % (SD)** | **Firmicutes, mean % (SD)** | **Proteobacteria, mean % (SD)** | **Bacteroides spp.,**  **mean % (SD)** | **Lactobacillus spp., mean % (SD)** | **Staphylococcus spp., mean % (SD)** |
| --- | --- | --- | --- | --- | --- | --- | --- | --- |
| Regular medication for asthma at the moment |  |  |  |  |  |  |  |  |
| Yes, N = 3 | 159 (174) | 12,068 (19,927) | 15% (26) | 31% (27) | 4.4% (6.6) | 14% (24) | 1.1% (1.5) | 6.6% (11) |
| No, N = 68 | 189 (127) | 12,343 (14,520) | 15% (21) | 36% (30) | 34% (35) | 13% (20) | 3.3% (11) | 14% (25) |
| Regular use of inhaled corticosteroids during the last year |  |  |  |  |  |  |  |  |
| Yes, N = 3 | 126 (109) | 877 (849) | 0.11% (0.13) | 27% (29) | 39% (37) | 0% (0) | 0.19% (0.21) | 11% (11) |
| No, N = 76 | 192 (130) | 13,442 (15,000) | 16% (22) | 37% (30) | 31% (35) | 14% (20) | 3.2% (11) | 14% (25) |
| Wheezing during last year |  |  |  |  |  |  |  |  |
| Yes, N = 13 | 157 (122) | 6855 (11,449) | 11% (18) | 33% (29) | 34% (34) | 8.9% (17) | 1.7% (3.9) | 15% (24) |
| No, N = 121 | 180 (128) | 15,414 (15,519) | 16% (22) | 42% (33) | 29% (34) | 13% (20) | 3.9% (14) | 13% (24) |
| Wheezing associated to respiratory infection before 2 years of age |  |  |  |  |  |  |  |  |
| Yes, N = 36 | 151 (112) | 11,892 (16,000) | 10% (18) | 35% (34) | 34% (36) | 8.1% (16) | 2.2% (9.7) | 17% (28) |
| No, N = 87 | 188 (130) | 15,158 (15,186) | 16% (21) | 43% (32) | 29% (34) | 13% (19) | 3.5% (12) | 12% (22) |
| Wheezing associated to respiratory infection during the last 2 years |  |  |  |  |  |  |  |  |
| Yes, N = 21 | 137 (120) | 9836 (13,450) | 15% (26) | 28% (30) | 29% (34) | 13% (25) | 1.2% (3.2) | 14% (24) |
| No, N = 98 | 191 (125) | 16,192 (15,732) | 16% (21) | 44% (32) | 30% (35) | 13% (19) | 3.1% (11) | 14% (25) |
| Physician visit due to respiratory infection associated wheezing |  |  |  |  |  |  |  |  |
| Yes, N = 35 | 158 (129) | 12,619 (15,903) | 12% (19) | 37% (34) | 27% (33) | 9.9% (18) | 2.4% (9.8) | 17% (30) |
| No, N = 94 | 184 (125) | 14,942 (15,284) | 16% (23) | 41% (32) | 31% (35) | 14% (21) | 3.2% (11) | 12% (21) |
| Medication for wheezing at physician’s visit |  |  |  |  |  |  |  |  |
| Yes, N = 30 | 148 (116) | 13,157 (16,547) | 10% (18) | 39% (35) | 27% (32) | 8.6% (17) | 2.4% (10) | 20% (32) |
| No, N = 99 | 186 (128) | 14,661 (15,141) | 16% (23) | 41% (32) | 31% (35) | 14% (21) | 3.3% (11) | 11% (21) |
| Dry cough at night, apart from a cough associated with infection during the last year |  |  |  |  |  |  |  |  |
| Yes, N = 11 | 131 (94) | 9813 (12,355) | 6.7% (14) | 49% (40%) | 32% (39) | 3.8% (12) | 10% (27) | 23% (27) |
| No, N = 122 | 183 (129) | 14,892 (15,566) | 16% (22) | 41% (32) | 29 (34) | 14% (21) | 3.1 (11) | 13 (24) |
| Wheezing during or after exercise during the last year |  |  |  |  |  |  |  |  |
| Yes, N = 4 | 181 (141) | 9425 (17,110) | 11% (23) | 33% (27) | 29% (36) | 10% (21) | 0.85% (1.3) | 7.9% (10) |
| No, N = 126 | 180 (127) | 14,708 (15,391) | 16% (22) | 41% (32) | 29 (34) | 13% (20) | 3.2% (11) | 14% (25) |

Number of participants with full data is lower (N = 134) than that of thoses variable used in the primary analysis, because the outcomes used in the primary analysis were actively collected with telephone contacts if families did not respond to the full survey

**Table S4.** Data regarding other variables in the questionnaire that are related to the diagnosis of cow’s milk allergy made by physicians used in the main primary analysis.

|  | **OTU’s (SD)** | **Number of reads (SD)** | **Bacteroidetes,**  **mean % (SD)** | **Firmicutes, mean % (SD)** | **Proteobacteria, mean % (SD)** | **Bacteroides spp.,**  **mean % (SD)** | **Lactobacillus spp., mean % (SD)** | **Staphylococcus spp., mean % (SD)** |
| --- | --- | --- | --- | --- | --- | --- | --- | --- |
| Cow’s milk allergy |  |  |  |  |  |  |  |  |
| Yes, diagnosis proven by oral food challenge,  N = 4 | 108 (89) | 406 (395) | 0.08% (0.12) | 9.2% (14) | 63% (44) | 0% (0) | 1.4% (2.9) | 4.7% (9.3) |
| No, N = 162 | 182 (126) | 15,088 (15,290) | 15% (22) | 43% (32) | 29% (33) | 13% (20) | 3.4% (11.4) | 14% (25) |
| Symptoms from cow’s milk allergy |  |  |  |  |  |  |  |  |
| Gastrointestinal, N = 5 | 147 (140) | 4,644 (8,062) | 9.6% (21) | 16% (15) | 34% (37) | 7.9% (18) | 9.2% (2.0) | 5.7% (8.4) |
| Skin symptoms, N = 2 | 229 (200) | 14,722 (14,722) | 20% (28) | 33% (36) | 46% (64) | 18% (25) | 4.1% (2.3) | 0.08% (0.12) |
| Both, N = 8 | 120 (137) | 5,953 (10,395) | 6.3% (18) | 22% (34) | 33% (45) | 5.7% (16) | 12% (31) | 1.5% (3.5) |
| Other allergic gastrointestinal symptoms |  |  |  |  |  |  |  |  |
| Yes, N = 8 | 185 (180) | 16,186 (16,411) | 16% (22) | 36% (35) | 22% (39) | 15% (21) | 12% (31) | 2.6% (6.7) |
| No, N = 122 | 178 (123) | 15,741 (15,512) | 15% (22) | 40% (32) | 30% (34) | 13% (20) | 3.2% (11) | 13% (23) |
| Other food allergy |  |  |  |  |  |  |  |  |
| Yes, N=10 | 159 (121) | 9,075 (11,267) | 11% (19) | 33% (34) | 34% (39) | 7.9% (17) | 9.4% (28) | 9.4% (21) |
| No, N=119 | 179 (128) | 16,556 (15,580) | 16% (22) | 41% (32) | 28% (34) | 13% (20) | 3.3% (11) | 14% (25) |

**Table S5.** Data regarding symptoms of allergic rhinitis symptoms inquired from the web-based questionnaire. Allergic rhinitis was not used in the primary analysis.

|  | **OTU’s (SD)** | **Number of reads (SD)** | **Bacteroidetes,**  **mean % (SD)** | **Firmicutes, mean % (SD)** | **Proteobacteria, mean % (SD)** | **Bacteroides spp.,**  **mean % (SD)** | **Lactobacillus spp., mean % (SD)** | **Staphylococcus spp., mean % (SD)** |
| --- | --- | --- | --- | --- | --- | --- | --- | --- |
| Allergic rhinits |  |  |  |  |  |  |  |  |
| Yes (diagnosis by a physician), N=8 | 238 (174) | 19,627 (15,793) | 22% (23) | 32% (24) | 20% (32) | 20% (21) | 1.8% (6.6) | 2.7% (4.2) |
| Yes (self noted), N=15 | 132 (95) | 6871 (12,289) | 3.5% (11) | 34% (36) | 46% (39) | 2.5% (9.6) | 23% (23) | 11% (22) |
| No, N=109 | 179 (126) | 16,664 (15,512) | 16% (23) | 43% (33) | 27% (33) | 13% (21) | 3.5% (12) | 15% (25) |
| Sneezing, or a runny, or a blocked nose when the child did not have a cold |  |  |  |  |  |  |  |  |
| Yes, N = 33 | 162 (128) | 11,528 (13,278) | 12% (23) | 36% (32) | 35% (37) | 11% (23) | 3.5% (15) | 12% (23) |
| No N=100 | 182 (126) | 17,122 (15,817) | 16% (22) | 43% (33) | 27% (33) | 13% (19) | 3.7% (12) | 14% (25) |
| Rhinitis accompanied by itchy-watery eyes during last year |  |  |  |  |  |  |  |  |
| Yes, N = 23 | 157 (128) | 12,900 (14,226) | 13% (25) | 37% (34) | 33% (37) | 11% (25) | 0.99% (1.6) | 11% (20) |
| No, N = 108 | 180 (126) | 16,206 (15,646) | 16% (21) | 41% (32) | 29% (34) | 13% (19) | 4.3% (14) | 14% (24) |
